# Fetomaternal Outcome in Severe Preeclamptic Women Undergoing Emergency Cesarean Section under Either General Or Spinal Anesthesia

**DOI:** 10.1155/2014/325098

**Published:** 2014-04-17

**Authors:** Suman Chattopadhyay, Ashok Das, Subrata Pahari

**Affiliations:** ^1^Department of Anaesthesiology, Medical College & Hospital, 88 College Street, Kolkata 700073, India; ^2^BC 103, Salt Lake, Kolkata 700064, India

## Abstract

This prospective observational study compared the effects of general and spinal anesthesia in 173 severe preeclamptic women undergoing emergency cesarean section. 146 (84.5%) patients underwent spinal anesthesia (SA) and 27 (15.5%) patients had general anesthesia (GA). Most of the patients were primigravid and nulliparous. Intraoperatively SA group required more intravenous fluid and vasopressor support, while GA group required more preoperative labetalol injection for blood pressure control. Overall 13.3% of patients required critical care, particularly GA group (44.4% versus 7.5%; *P* < 0.001). Patients receiving GA had a higher mortality (25.9% versus 1.4%; *P* < 0.001). The length of hospital stay was comparable. Significantly more neonates of patients receiving GA were found to be preterm (77.8% versus 44.5%; *P* < 0.01) and required advanced resuscitation. GA group also had higher neonatal mortality (29.6% versus 11%; *P* < 0.05). To conclude, severe preeclamptic mothers receiving general anesthesia and their babies required more critical care support. Maternal as well as neonatal mortality was significantly higher with general anesthesia.

## 1. Introduction

Severe preeclampsia is the development of hypertension characterised by systolic blood pressure exceeding 160 mm Hg and/or diastolic blood pressure exceeding 110 mm Hg, together with proteinuria (>5 gm/24 hr) after 20 weeks of gestation. It can be accompanied by symptoms or signs of imminent eclampsia, pulmonary edema, or HELLP (hemolysis, elevated liver enzymes, and low platelet count) syndrome [1]. Fetal complications include placental abruption, intrauterine growth restriction, premature delivery, and intrauterine fetal death. The incidence of stillbirths and neonatal deaths in mothers who suffered severe preeclampsia was 22.2/1000 and 34.1/1000, respectively, in the UK with a higher incidence in developing countries [2]. The impact of the disease is felt more severely in developing countries where, unlike other more prevalent causes of maternal mortality (such as hemorrhage and sepsis), medical interventions may be ineffective due to late presentation of cases [3]. The problem is confounded by the continued mystery of the etiology and the unpredictable nature of the disease [3]. Preeclampsia is a principal cause of fetal morbidity and mortality, also the leading reason of maternal ICU admissions, and responsible for 15–20% of maternal deaths worldwide [4].

In India the incidence of preeclampsia is 7.6% during pregnancy of which 3.3% is severe preeclampsia [5]. Delivery of the infant and placenta is the only effective treatment. Delivery at an earlier gestational age, however, is associated with an increased risk of adverse neonatal outcome [6]. Women with preeclampsia have an increased rate of cesarean section consequent upon the high incidence of intrauterine growth restriction, fetal distress, and prematurity [6]. Cesarean section on the other hand increases the risk of cardiopulmonary morbidity associated with preeclampsia [7]. This is due to the altered hemodynamics in women with preeclampsia, particularly in an emergent situation. This risk is present with both spinal and general anesthesia and continues to challenge anesthetists worldwide.

The risk of general anesthesia (GA) is significantly increased in obstetric population. The incidence of failed intubation and aspiration are eight times higher than nonobstetrical patient [8]. Other associated risks are systemic and pulmonary hypertension, which may be deleterious in this group of patients [7]. Moreover, the universal use of magnesium sulphate in severe preeclamptic patients prolongs the duration of muscle relaxants making the duration and recovery from GA in these patients unpredictable [6]. Spinal anesthesia (SA) is generally chosen, especially if anesthetic resources are limited, as in developing countries. The quality of anesthesia can be superior with SA and requires less equipment and training compared to epidural anesthesia.

However some recent studies have highlighted increased incidence of fetal acidosis with SA, particularly with use of vasopressors to treat hypotension [9]. In severe preeclampsia, particularly with a diastolic pressure ≥ 110 mm Hg, the fetus is compromised due to greater neonatal base deficit [10]. GA, as well as regional anesthesia, has been shown to be acceptable and safe methods for conducting cesarean deliveries in preeclampsia, if steps are taken to ensure a careful approach to either technique [6]. The hemodynamic alterations produced by SA are comparable with that in GA in severe preeclampsia [11].

In this scenario where there is still a dilemma about whether SA or GA is better for parturient with severe preeclampsia, our study primarily studied the effects of anesthetic technique on the fetomaternal outcome (if any) in severe preeclamptic patients undergoing cesarean section in the setup of a tertiary care teaching hospital in a developing country. The secondary endpoints of this study are to study the demographic profile, obstetric parameters, and perioperative usage of antihypertensives, intravenous fluids, and vasopressors among others.

## 2. Methods 

This institution based prospective, observational study was carried out after approval of hospital ethics committee and informed patient consent. All severe preeclamptic patients admitted in Medical College & Hospital, Kolkata, at 34 weeks or more gestational age between February 2012 and June 2013, undergoing emergency cesarean section, and not conforming to any of the exclusion criteria, were included in the current study. 

**Figure 1:**
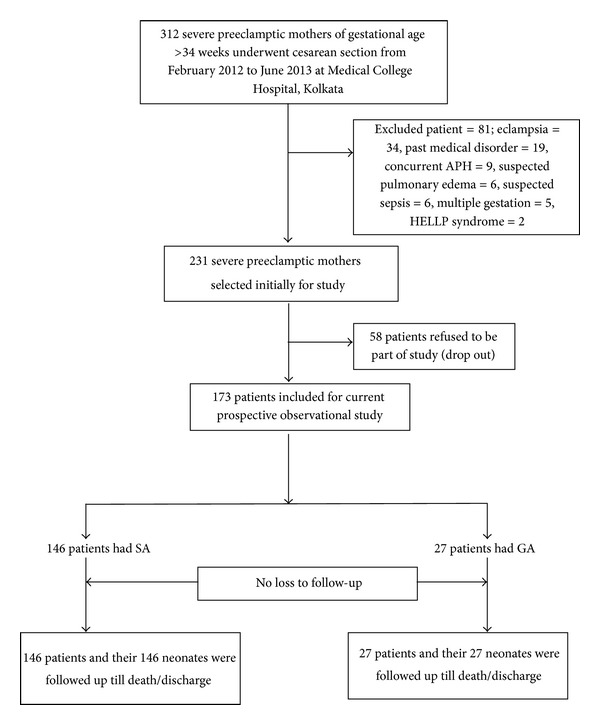


The exclusion criteria in this study were as follows: eclamptic patients; patients having previous history of medical disorders like chronic hypertension, diabetes, connective tissue disorder, thyroid dysfunction, epilepsy, renal disease, heart disease, and obesity; patients having any severe allergic reaction; patients having abruptio placenta or placenta previa; patient having coagulopathy, thrombocytopenia with platelet count less than 80,000/cm^3^, sepsis, neurological problems, hypovolemia, or pulmonary edema; multiple gestations or any congenital anomalies of new born baby. See Figure 1. 

Patients with a diagnosis of severe preeclampsia were admitted in the ward or labour room for emergency cesarean section and initial obstetric management was given according to existing hospital protocol. Thereafter general, physical, abdominal, and pelvic examinations were done. Initial investigations like complete hemogram, absolute platelet count, liver function tests, serum creatinine, and urine dipstick for grading of proteinuria were performed after admission and twice weekly thereafter. The latest reports before performance of the cesarean section were only included for the purpose of the study. Urine output measurement was done by Foley's catheterisation inserted after anesthesia procedure.

The study started after a severely preeclamptic parturient underwent anesthesia given by an anesthesiologist not involved in the study, as per personal choice and expertise and following institutional protocol. Overall 19 staff anesthesiologists performed anesthesia in various shifts in the obstetric emergency OT of our hospital during the study period. For SA, inj. hyperbaric bupivacaine (0.5%), 10 to 15 mg was given intrathecally with or without 20 to 25 mcg of fentanyl. For GA, inj. propofol 1.5 to 2.5 mg·kg^−1^ with inj. suxamethonium, 1 to 2 mg·kg^−1^ i.v. was given for rapid sequence intubation and maintained with isoflurane 0.3 to 1.5 MAC as required. Muscle relaxants were excluded whenever possible, as their effect is unpredictably prolonged with preoperative magnesium sulphate (MgSO_4_) therapy. The intraoperative analgesics varied from fentanyl 1 to 2 mcg·kg^−1^ i.v. with or without other nonopioid analgesics like paracetamol 1 g infusion i.v. or diclofenac 75 mg as i.v. infusion. In case of failure of SA to provide sufficient anesthesia, GA was given to the patient.

The study included a detailed follow-up of the same mothers who underwent cesarean section and their respective babies till discharge or death during the period of hospital stay. The mothers were followed up from cesarean section till their discharge or in-hospital death during current admission. Any admission/intervention requiring critical care support was noted. The newborn babies were observed as to their condition at delivery and subsequent requirement for any specialized neonatal intensive care in the Sick Neonatal Care Unit (SNCU). This study did a follow-up of these babies till they were bonded to their mothers or till any neonatal mortality.

The study also observed several other parameters of the patients and their babies. These parameters included the demographic profile, indications of cesarean section in severe preeclampsia, obstetric parameters, perioperative hemodynamic status, grade of proteinuria, perioperative use of antihypertensives, and MgSO_4_ among others. Apart from the type of anesthesia administered the study also looked at the perioperative use of intravenous fluids, skin incision to delivery (I-D) interval, uterine incision to delivery (U-D) interval, oxytocin dosage, duration of surgery, and anesthesia.

For seizure prophylaxis, MgSO_4_ was given to all patients diagnosed with severe preeclampsia as per hospital protocol. For controlling blood pressure, booked mothers were given methyldopa regularly (500 mg thrice daily, 1500 mg); however, those not still controlled (diastolic blood pressure > 110 mm Hg) or emergency unbooked cases received injection labetalol. If any derangement in the mother's general conditions occurred, then mothers were shifted to critical care unit (CCU) for better management. Incidences and causes of morbidity and mortality were followed up in those patients requiring critical care. The length of hospital stay was noted in all patients who survived.

After delivery of the baby, initial resuscitation was done. Newborn babies were assessed regarding the term, gestational age, Apgar score at 1 and 5 minutes, heart and respiratory rate after resuscitation, the amount of resuscitative efforts given (in terms of drying, suction, and any requirement of oxygenation or bag mask ventilation) and the neurobehavioral assessment was done by the New Ballard Score (NBS) [12]. Babies were sent to the SNCU nursery for close observation, monitoring of vitals, and supportive management for first 24 hrs. while mothers of these babies were kept in a separate ward for observation and MgSO_4_ therapy as per hospital protocol. In the nursery signs of respiratory distress and need for respiratory support were noted. In situations where there was any subsequent requirement of neonatal critical care, the cause of the admission and mortality (if any) was noted. The time to initiate breast feeding in these newborns was also noted.

Data was entered as per case record form particularly designed for this study for relevant statistical methods. Results were directed to establish the specific objectives of the study. Categorical variables were expressed as number of patients and percentage (%) of patients and compared across the groups using Pearson's Chi Square test for independence of attributes. Continuous variables are expressed as mean ± standard deviation and compared across the 2 groups using unpaired* t-*test. The results were collected in Microsoft Excel for analysis with statistical software SPSS version 16. An alpha level of 5% has been taken; that is, if any *P* value is less than 0.05 it has been considered as being of significance.

## 3. Results

Of the 173 patients of severe preeclampsia undergoing emergency cesarean section 146 (84.5%) patients were administered spinal anesthesia (SA) and 27 (15.5%) patients received general anesthesia (GA) in our study as per choice of anesthesiologists concerned. Of the various indications, a very high percentage of patients underwent GA due to fetal distress (37% versus 16.4%; *P* < 0.05). All other indications for cesarean section were similar in-between the patients who underwent spinal and general anesthesia (Table 1).

The demographic profiles of the two groups were comparable in terms of age, weight, and height (Table 2). Patients given GA had more incidence of fetal distress and had earlier cesarean sections with lesser gestational age compared to patients given spinal anesthesia (*P* < 0.05).

In our study 54.9% of women had come from urban areas, while 45.1% from rural areas. Most of the mothers were literate and booked (96.5% and 94.2%, resp.), and 85.5% came from a low socioeconomic status. Around 69% were primigravida and 71% were nulliparous.

A vast majority of severe preeclamptic patients (98.8%) had severe proteinuria (grade 3+ or more). Of them 69.4% had a grade 3+ proteinuria, and 29.4% had a grade 4+ proteinuria. The average preoperative platelet count was below 1,00,000/mm^3^ in both SA and GA groups (97,610.27 ± 23,001.04 versus 89,688.89 ± 19,599.34; *P* > 0.05).

Both groups received similar amounts of preinduction Ringer's Lactate (RL) solution preoperatively (552.4 ± 128.3 and 516.67 ± 146.1 mL resp.). The preinduction hemodynamic parameters in terms of systolic, diastolic blood pressure, and pulse rate were similar between groups.

Overall, blood pressure of 51.4% mothers was controlled with methyldopa alone, while 48.6% required a further dose of inj. labetalol. More patients in the GA group required inj. labetalol (88.9% versus 48.6%, *P* < 0.001) to control blood pressure perioperatively. This is shown in Table 3. All patients received magnesium sulphate as antiseizure prophylaxis.

Intraoperatively more Ringer's Lactate (RL) fluid and vasopressors were given in the spinal group (*P* < 0.001 and *P* < 0.01 resp.). Skin incision to baby delivery (I-D) interval as well as the uterine incision to baby delivery (U-D) interval was similar in both groups. The overall duration of surgery and duration of anesthesia were comparable for spinal and general anesthesia. Oxytocin requirements were also similar in both groups (Table 4).

Maternal complications in order of frequency were pain at the spinal site (8%), pulmonary edema, headache, and uncontrolled blood pressure (5.2% each). Subgroup analysis shows that 22.2% of patients given GA developed pulmonary edema and 14.8% in this group had uncontrolled hypertension. Pain at the spinal site was the most common complication with SA (9.6%). Two patients of SA group had developed paresthesia in right leg postoperatively and one patient developed cerebrovascular accident (CVA). Other complications included postoperative vomiting and wound gaping (Table 5). 62.3% of patients in the SA group and 33.3% in the general anesthesia group were free of any complications (*P* < 0.05). However problems like headache, vomiting, fever, pain at injection site, paresthesia, and visual disturbance occurred only in patients given SA.

In our study 87.1% of the severe preeclamptic mothers were directly shifted to ward, while 13.3% required critical care. Overall 44.4% of patients in GA group required critical care at any point of time compared to 6.3% in SA group. During the follow-up period in the ward one patient developed CVA and was shifted to neuromedicine ward (Table 6).

**Figure 2:**
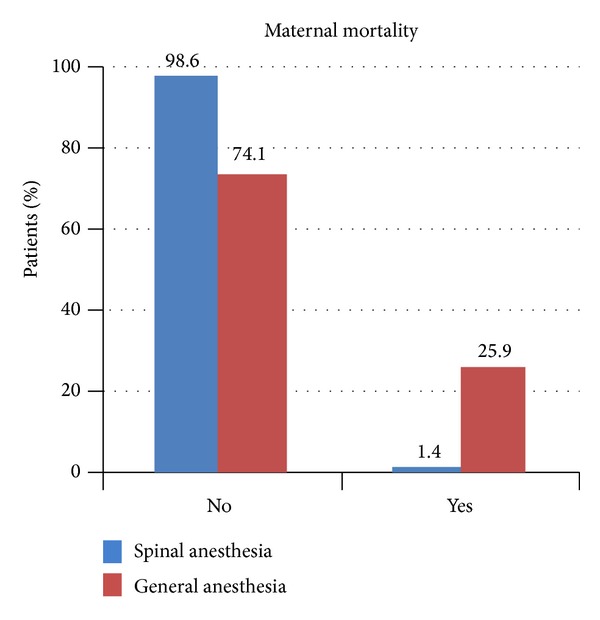
Maternal mortality comparisons. GA group patients had higher mortality.

Overall 9 mothers died. Of these 7 patients (25.9%) were given GA and 2 patients (1.4%) were given SA (*P* < 0.001). This is depicted in Figure 2.

The common causes of maternal mortality were acute renal failure (ARF), disseminated intravascular coagulation (DIC), congestive cardiac failure (CCF), HELLP syndrome, and sepsis. Intergroup comparisons showed that the causes of mortality did not differ statistically among the two groups (Table 7).

Preterm delivery was higher in GA group (77.8% versus 44.5%; *P* < 0.05). Weight of babies was similar in both groups. Neurobehavioral assessment of babies performed with the New Ballard Score showed similar maturity of the newborns in the two groups. Apgar score at 1 minute was lower in patients given GA (7.66 ± 1.27 versus 6.52 ± 1.16 minute; *P* < 0.001), but 5-minute Apgar scores were similar in both groups. Heart rate after initial resuscitation was higher in babies of the GA group (*P* < 0.05). Respiratory rate after initial resuscitation and initiation time of breast feeding were similar in both groups (Table 8).

Initial resuscitation with suctioning and drying was performed on all newborns.  Table 9 denotes the amount of resuscitation performed in terms of drying; suction and drying; suction/drying/oxygenation; or requirement of bag and mask ventilation. More babies whose mothers had received GA required supplemental oxygen and bag mask ventilation (*P* < 0.001).

**Figure 3:**
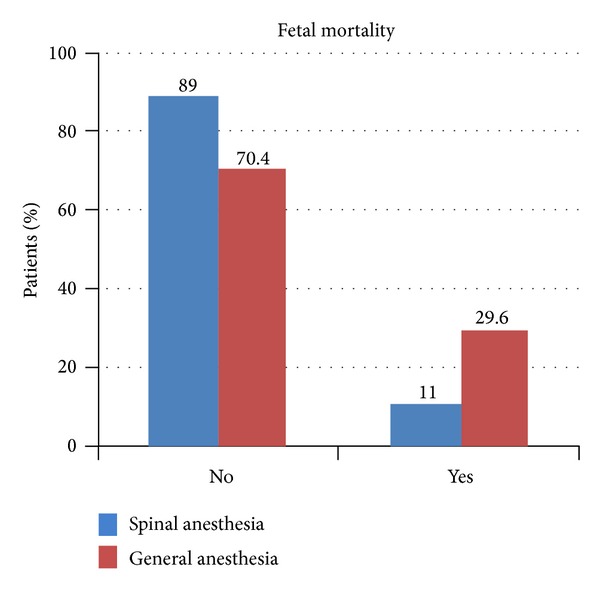
Fetal mortality comparisons. GA was associated with higher fetal deaths.

Of all the 24 babies who died 8 (29.6%) were from the GA group and 16 (11%) were from the spinal group. Intergroup comparison shows that fetal mortality was significantly higher in GA group (*P* < 0.01), Figure 3.

The causes of fetal mortality like hypoxic ischemic encephalopathy (HIE), ventilator associated pneumonia (VAP), sepsis, aspiration pneumonia, meconium aspiration syndrome (MAS), and hyperbilirubinemia with sepsis were comparable in both groups (*P* > 0.05%). The main causes of neonatal mortality were HIE (47.7%) and sepsis (37.5%) (Table 10).

The overall duration of hospital stay did not differ (7.7 ± 2.37 versus 8.59 ± 2.97 days, *P* > 0.05).

## 4. Discussions

In the present study 146 patients were administered spinal anesthesia and 27 patients received general anesthesia for emergency cesarean section with a diagnosis of severe preeclampsia. These patients were predominantly young, educated but poor. A vast majority of patients were primigravid. All patients had significant proteinuria and an average platelet count slightly below 1,00,00/cmm. Patients in the GA group had more incidences of fetal distress and their babies were more premature in our study.

Intraoperatively, significantly higher number of patients having GA required additional preoperative and intraoperative labetalol injection. Intraoperatively more Ringer's Lactate (RL) fluid and vasopressors were given in the spinal group (*P* < 0.05). Operative parameters like skin incision to baby delivery (I-D) and uterine incision to delivery (U-D) intervals were similar as also the oxytocin use after delivery of the baby. The duration of surgery and anesthesia were also comparable in the two groups.

Postoperatively, patients receiving GA required more critical care support. A disproportionately higher number of maternal deaths were from the group given GA. The incidence of preterm delivery was more under GA. The babies of mothers receiving GA required advanced resuscitation in the form of supplemental oxygen and bag mask ventilation in more number of cases. A significantly higher population of babies in GA group died.

This preponderance of spinal anesthesia as the preferred anesthetic technique for cesarean section in patients with severe preeclampsia by anesthetists is similar to another recent retrospective analysis in similar cohorts of patient performed by Keerath and Cronje [13]. In their setup in a teaching hospital in South Africa only 25% of patients received GA. The obstetric findings were similar to an Indian study performed by Singhal et al. [14]. A recent retrospective analysis by Ajuzieogu et al. [15] from another third world setting also found similar maternal obstetric parameters. A systematic review by Douglas [16] proposed a platelet count threshold of 80,000/mm^3^ as adequate for the administration of neuraxial anesthesia in pregnant women without other risk factors. The average platelet counts in both our groups were much higher.

This increased requirement of intravenous antihypertensive medication in the general anesthesia group may be due to stress of laryngoscopy, intubation, and increased catecholamine release [6]. These findings were similar to a prospective randomised control study by Ahsan-ul-Haq [17].

Maternal complications which required critical care support were pulmonary edema, acute renal failure, convulsion, DIC, headache, postpartum hemorrhage, HELLP syndrome, visual disturbance, lower respiratory tract infection, and congestive cardiac failure. These findings were similar to an Indian study performed by Singhal et al. [14].

However, patients receiving general anesthesia required significantly more critical care support. These findings were similar to the studies by Ahsan-ul-Haq [17] and Okafor et al. [18]. The former was a prospective study, while the latter was a retrospective study which compared the patients of severe preeclampsia in two third world countries. Both studies found a disproportionately higher maternal mortality in patients receiving general anesthesia compared to spinal anesthesia in severe preeclamptic patients. On the contrary, Ajuzieogu et al. [15] in their retrospective analysis found maternal mortality were similar with spinal and general anesthesia. The total length of stay (LOS) in hospital did not differ between the groups in our study. This was similar to findings by Fassoulaki et al. [19] in their retrospective analysis of anesthesia on the LOS.

The babies of mothers receiving general anesthesia required advanced resuscitation in the form of supplemental oxygen and bag mask ventilation in more number of cases (*P* < 0.05). Ajuzieogu et al. [15] have found significant birth asphyxia in babies of severe preeclamptic mothers receiving general anesthesia compared to spinal anesthesia. Dasgupta et al. [20] found that neonatal umbilical artery base deficit was significantly higher in GA group and that these neonates required more resuscitative efforts, a finding similar to our studies. Ahsan-ul-Haq [17] also found lower 1-minute Apgar score with GA in a similar cohort given general and spinal anesthesia. 

All the babies were shifted to Sick Neonatal Care Unit (SNCU) of our hospital as per protocol. A significantly higher population of babies in the general anesthesia group died. The poor neonatal outcome of babies of severe preeclamptic mothers receiving general anesthesia in our study is shared by other studies like Keerath and Cronje [13] and Okafor et al. [18]. However, not all studies held a similar view. Ajuzieogu et al. [15], Moodley et al. [21], and Santos and Birnbach [22] found similar neonatal outcomes irrespective of anesthesia administered. Laudenbach et al. [23] on the other hand noted a higher risk of mortality in neonates in the spinal group compared to general and epidural anesthesia.

The optimal anesthetic technique for cesarean delivery in severely preeclamptic women remains controversial. General and regional anesthesia are equally acceptable for cesarean delivery in pregnancies complicated by severe preeclampsia if steps are taken to ensure a careful approach to either method. Given that failure to vasodilate is a common factor in preeclampsia, neuraxial blockade during labor and delivery appears to be a logical choice if patient is stable with a normal level of consciousness and no neurological deficits [6]. Advantages include (1) provision of high-quality analgesia, which attenuates the hypertensive response to pain, (2) a reduction in levels of circulating catecholamines and stress-related hormones, (3) possible improvement in intervillous blood flow, thus obviating the need for general anesthesia with its attendant risks.

There are several reasons for preferring spinal anesthesia for cesarean sections. Babies born to mothers having spinal anesthesia may be more alert and less sedated as they have not received any general anesthetic agents through the placental circulation [15]. As the mother's airway is not compromised, there is a reduced risk of aspiration of gastric contents causing chemical pneumonitis. The onset of block is faster and quality of anesthesia is generally superior with spinal anesthesia and requires less equipment and training and more importantly less time to perform compared to epidural and combined spinal epidural anesthesia, particularly in an emergency setting [17]. The small doses of local anesthetics required to perform spinal anesthesia also reduce the risks of systemic toxicity compared to epidural and combined spinal epidural anesthesia.

Our study suffered from several limitations. As it was a prospective observational cohort study there were no randomizations of the groups regarding anesthesia technique. In fact, due to choice of anesthesia being left to the sole discretion of the concerned anesthetist, it was apparent that only 15.5% of the patients having severe preeclampsia received general anesthesia.

In our study over 17 months only 27 patients of severe preeclampsia included in the present study received general anesthesia. This small sample size of 27 patients of the GA group is a hindrance to draw definite conclusions regarding the poor fetomaternal outcome. However, a recent retrospective study by Keerath and Cronje [13] has drawn conclusions from 21 patients undergoing GA under similar situations in a similar setup. It is also necessary to point out that, of the 81 patients of severe eclampsia excluded from this study, 52 patients having eclampsia, sepsis, pulmonary edema, antepartum hemorrhage, and HELLP syndrome had undergone GA.

The presenting symptoms of severe preeclampsia could not be ascertained in most of the cases due to poor observation in the case records files and thus left from the purview of our observation. Other areas of poor documentation were quantification of fluids, particularly colloids as well as use of vasopressors. Preoperative management of blood pressure was not standardized.

Also there is no uniform expertise or technique followed by anesthesiologists while performing anesthesia on patients of severe preeclampsia. There was great variation of the use of anesthetic drugs, particularly in the general anesthesia group. Critical care management of mothers and neonates may not have been consistent and may be related to poor outcomes.

Lastly, patients in the general anesthesia group had more incidence of fetal distress and had significantly lower gestational age. This may have ultimately led to poorer outcome in neonates in the general anesthesia group. Also, the fetal acid base status could not be done in our setup, which is also a significant limitation of this study.

To conclude, severe preeclamptic mothers receiving general anesthesia and their babies required more critical care support. Maternal as well as neonatal mortality was significantly higher with general anesthesia. Spinal anesthesia is a safer alternative to general anesthesia in severe preeclampsia with less postoperative morbidity and mortality.

## Figures and Tables

**Table 1:** Indication of cesarean section in severe preeclampsia.

Indication of cesarean section	GROUP		Total	*P* value	Significance
	Spinal anesthesia	General anesthesia			
Severe preeclampsia	71 (48.6%)	10 (37%)	81 (46.8%)	0.254	Not Significant
Fetal distress	24 (16.4%)	10 (37%)	34 (19.7%)	**0.035**	**Significant**
Unfavourable cervix	5 (3.4%)	1 (3.7%)	6 (3.5%)	0.943	Not significant
Less fetal movement	8 (5.5%)	2 (7.4%)	10 (5.8%)	0.720	Not significant
Contracted pelvis	8 (5.5%)	1 (3.7%)	9 (5.2%)	0.664	Not significant
Post cesarean section	16 (11%)	1 (3.7%)	17 (9.8%)	0.104	Not significant
IUGR/ oligohydramnios	6 (4.1%)	1 (3.7%)	7 (4%)	0.919	Not significant
Induction failure	3 (2.1%)	1 (3.7%)	4 (2.3%)	0.666	Not significant
Premature rupture of membrane	3 (2.1 %)	0 (0 %)	3 (1.2%)	0.154	Not significant
High floating head	2 (1.4%)	0 (0 %)	2 (1.2%)	0.154	Not significant
Obstructed labour	1 (0.7%)	0 (0 %)	1 (0.6%)	0.316	Not significant

Total	146 (100%)	27 (100%)	173	**Pearson's Chi Square test**	

**Table 2:** Demographic profile of mothers regarding age, weight, height, and gestational age.

Parameters	GROUP		*P* value	Significance
	Spinal anesthesia	General anesthesia		
	Mean ± std. deviation	Mean ± std. deviation		
Age	23.42 ± 4.32	22.78 ± 4.91	0.490	Not significant
Weight	63.79 ± 3.12	64.48 ± 3.01	0.292	Not significant
Height	156.47 ± 3.14	156.78 ± 3.11	0.639	Not significant
Gestational age	35.58 ± 2.23	34.63 ± 1.5	**0.036**	**Significant**

Student unpaired *t*-test.

**Table 3:** Antihypertensive and antiseizure therapy in antenatal, preoperative, and intraoperative period.

Antihypertensive and antiseizure therapy	GROUP		Total	*P* value	Significance
	Spinal anesthesia	General anesthesia			
Labetalol	65 (44.5%)	24 (88.9%)	89 (51.4%)	**<0.001**	**Significant**
Methyldopa	81 (55.5%)	3 (11.1%)	84 (48.6%)		
Magnesium sulphate	146 (100%)	27 (100 %)	173 (100%)	>0.05	Not significant

Total	146 (100%)	27 (100%)	173 (100%)	**Pearson's Chi Square test**	

**Table 4:** Intraoperative hemodynamic, operative, and anesthesia parameters.

Intraoperative parameters	GROUP		*P* value	Significance
	Spinal anesthesia	General anesthesia		
	Mean ± std. deviation	Mean ± std. deviation		
Intraoperative systolic BP	128.36 ± 12.27	133.19 ± 10.16	0.056	Not significant
Intraoperative diastolic BP	78.55 ± 10.9	84.52 ± 9.7	**0.009**	**Significant**
Intraoperative IV RL bottles	3.01 ± 0.49	2.48 ± 0.51	**<0.001**	**Significant**
Vasopressor use	76 (52.1%)	9 (33.3%)	**<0.01**	**Significant**
Skin incision to delivery (minute)	4.58 ± 1.43	4.22 ± 1.46	0.239	Not significant
Uterine incision to delivery (sec)	61.56 ± 17.98	55.96 ± 5.93	0.112	Not significant
Duration of surgery (minute)	49.54 ± 6.72	49.15 ± 7.03	0.782	Not significant
Duration of anesthesia (minute)	56.64 ± 14.21	59.63 ± 7.2	0.287	Not significant
Oxytocin in unit	13.32 ± 2.51	13.89 ± 2.12	0.272	Not significant

Student's unpaired *t*-test and Pearson's Chi Square test.

**Table 5:** Comparison of maternal complications in two groups.

Complications	GROUP		Total (%)
	Spinal anesthesia	General anesthesia	
Nil	91 (62.3%)	9 (33.3%)	57.8%
Headache	9 (6.2%)	0	5.2%
Vomiting	3 (2.1%)	0	1.73%
Fever and wound gaping	3 (2.1%)	0	1.73%
High blood pressure	5 (3.4%)	4 (14.8%)	5.2%
Pain at spinal injection side	14 (9.6%)	0	8%
Paresthesia	2 (1.4%)	0	1.15%
Visual disturbance	3 (2.1%)	0	1.73%
Convulsion	3 (2.1%)	2 (7.4%)	2.9%
Acute renal failure (ARF)	4 (2.8%)	2 (7.4%)	3.46%
Pulmonary edema	3 (2.1%)	6 (22.2%)	5.2%
Postpartum hemorrhage (PPH)	2 (1.4%)	1 (3.7%)	1.73%
Disseminated intravascular coagulation (DIC)	1 (0.7%)	2 (7.4%)	1.73%
HELLP syndrome	0	1 (3.7%)	0.57%
Cerebrovascular accident (CVA)	1 (0.7%)	0	0.57%
Lower respiratory tract infection (LRTI)	1 (0.7%)	0	0.57%
Congestive cardiac failure (CCF)	1 (0.7%)	0	0.57%

**Table 6:** Postoperative maternal admission to the critical care unit (CCU).

Ward	GROUP		Total	*P* value	Significance
	Spinal anesthesia	General anesthesia			
General ward	134 (93.1%)	15 (55.6%)	149 (87.1%)	**<0.001**	**Significan**t
CCU	11 (7.5%)	12 (44.4%)	23 (13.3%)		
Neuromedicine ward	1 (0.7%)	0 (0)	1 (0.6%)		

Total	146 (100%)	27 (100%)	173 (100%)	**Pearson's Chi Square test**	

**Table 7:** Distribution of causes of maternal mortality in the two groups.

Causes of maternal mortality	GROUP		Total	*P* value	Significance
	Spinal anesthesia	General anesthesia			
ARF	1 (50%)	2 (28.6%)	3 (33.3%)	0.864	Not significant
DIC	1 (50%)	2 (28.6%)	3 (33.3%)		
CCF	0	1 (14.3%)	1 (11.1%)		
HELLP syndrome	0	1 (14.3%)	1 (11.1%)		
Sepsis	0	1 (14.3%)	1 (11.1%)		

Total	2	7	9	**Chi Square test**	

**Table 8:** Comparison of fetal parameters at delivery in the two groups.

Fetal parameters	GROUP		*P* value	Significance (Student's unpaired *t*-test)
	Spinal anesthesia	General anesthesia		
	Mean ± SD	Mean ± SD		
Weight (kg)	2.48 ± 0.56	2.28 ± 0.43	0.090	Not Significant
Preterm	65 (44.5%)	21 (77.8%)	**0.001**	**Significant**
New Ballard Score ( weeks)	35.07 ± 1.84	34.59 ± 1.55	0.208	Not Significant
Apgar score at 1 minute	7.66 ± 1.27	6.52 ± 1.16	**<0.001**	**Significant**
Apgar score at 5 minutes	8.79 ± 0.65	8.59 ± 0.93	0.172	Not Significant
Heart rate/minute	142.36 ± 12.53	151.48 ± 10.45	**<0.001**	**Significant**
Respiratory rate/minute	41.25 ± 6.9	38.85 ± 6.03	0.093	Not Significant
Breast feeding after cesarean (hrs.)	29.01 ± 21.73	35.56 ± 41.1	0.225	Not Significant

**Table 9:** Comparison between two groups regarding degree of resuscitation.

Degree of resuscitation	GROUP		Total	*P* value	Significance
	Spinal anesthesia	General anesthesia			
Drying	30 (20.5%)	2 (7.4%)	32 (18.5%)	**<0.001**	**Significant**
Suction and drying	84 (57.5%)	10 (37%)	94 (54.3%)		
Supplemental oxygen	13 (8.9%)	10 (37%)	23 (13.3%)		
Bag and mask ventilation	19 (13%)	5 (18.5%)	24 (13.9%)		

Total	146 (100)	27 (100)	173 (100)		

Pearson's Chi Square test.

**Table 10:** Comparison of causal relationship of fetal mortality.

Causes of neonatal mortality	GROUP		Total	*P* value	Significance
	Spinal anesthesia	General anesthesia			
HIE-3	6 (37.5%)	1 (12.5%)	7 (29.2%)	0.119	Not significant
HIE-4	2 (12.5%)	1 (12.5%)	3 (12.5%)		
VAP	1 (6.2%)	0	1 (4.2%)		
Sepsis	7 (43.8%)	2 (25%)	9 (37.5%)		
Aspiration pneumonia	0	1 (12.55%)	1 (4.2%)		
MAS	0	1 (12.5%)	1 (4.2%)		
Sepsis and hyperbilirubinemia	0	2 (25%)	2 (8.3%)		

Total	16 (100)	8 (100)	24 (100)	**Pearson's Chi Square test**	
